# Antiviral effect of SARS-CoV-2 N-specific CD8^+^ T cells induced in lungs by engineered extracellular vesicles

**DOI:** 10.1038/s41541-023-00686-y

**Published:** 2023-06-02

**Authors:** Francesco Manfredi, Chiara Chiozzini, Flavia Ferrantelli, Patrizia Leone, Katherina Pugliese, Massimo Spada, Antonio Di Virgilio, Andrea Giovannelli, Mauro Valeri, Andrea Cara, Zuleika Michelini, Mauro Andreotti, Maurizio Federico

**Affiliations:** 1 National Center for Global Health, Istituto Superiore di Sanità, Viale Regina Elena, 299, 00161 Rome, Italy; 2 National Center for Animal Experimentation and Welfare, Istituto Superiore di Sanità, Viale Regina Elena, 299, 00161 Rome, Italy

**Keywords:** Vaccines, Infectious diseases

## Abstract

Induction of effective immunity in the lungs should be a requisite for any vaccine designed to control the severe pathogenic effects generated by respiratory infectious agents. We recently provided evidence that the generation of endogenous extracellular vesicles (EVs) engineered for the incorporation of Severe Acute Respiratory Syndrome Coronavirus (SARS-CoV)-2 Nucleocapsid (N) protein induced immunity in the lungs of K18-hACE2 transgenic mice, which then can survive the lethal virus infection. However, nothing is known about the ability of the N-specific CD8^+^ T cell immunity in controlling viral replication in the lungs, a major pathogenic signature of severe disease in humans. To fill the gap, we investigated the immunity generated in the lungs by N-engineered EVs in terms of induction of N-specific effectors and resident memory CD8^+^ T lymphocytes before and after virus challenge carried out three weeks and three months after boosting. At the same time points, viral replication extents in the lungs were evaluated. Three weeks after the second immunization, virus replication was reduced in mice best responding to vaccination by more than 3-logs compared to the control group. The impaired viral replication matched with a reduced induction of Spike-specific CD8^+^ T lymphocytes. The antiviral effect appeared similarly strong when the viral challenge was carried out 3 months after boosting, and associated with the persistence of N-specific CD8^+^ T-resident memory lymphocytes. In view of the quite low mutation rate of the N protein, the present vaccine strategy has the potential to control the replication of all emerging variants.

## Introduction

Several vaccines were developed and distributed within an unprecedentedly short time in response to the spread of Severe Acute Respiratory Syndrome Coronavirus (SARS-CoV)-2. All vaccine preparations were conceived to elicit anti-Spike protein immune responses, and their effectiveness relies on the generation of neutralizing antibodies. Administration of anti-SARS-CoV-2 mRNA-based vaccines leads to the production of extraordinarily high levels of anti-Spike antibodies in serum^1,2^. Regrettably, however, in humans the generation of Spike-binding antibodies at the viral port of entry, i.e., the mucosa of the upper respiratory tract, does not result in a significant virus neutralization activity^3–7^. In addition, anti-Spike immunity in lungs of vaccinees was found either barely detectable or absent^8^.

The virtual absence of Spike-specific cell immunity in lungs of vaccinees is not surprising given that the vaccine is administrated intramuscularly. In fact, it is known that the development of cell immunity in pulmonary tissues is largely independent of the events occurring in both peripheral circulation and distal lymphoid organs. Lymphocytes in lungs are maintained separately from the pool of circulating lymphocytes, and their continuous loss through intraepithelial migration toward the airways is constantly replenished by homeostatic proliferation^9^. In this scenario, the identification of new anti-SARS-CoV-2 vaccines eliciting adequate antiviral immunity in lungs would be of outmost relevance.

All cell types constitutively release nanovesicles (collectively referred to as extracellular vesicles, EVs), which are key players in intercellular communication^10^. We developed a CD8^+^ T-cell-based vaccine platform based on intramuscular (i.m.) injection of a DNA vector coding for antigens of interest fused at the C-terminus of a biologically inactive Human Immunodeficiency Virus (HIV)-Type 1 Nef protein (Nef^mut^) having an unusually high efficiency of incorporation into EVs. This unique feature is maintained even when foreign polypeptides are fused to its C-terminus^11,12^. Both N-terminal myristoylation and palmitoylation fasten Nef^mut^ to the luminal membrane leaflets, thus allowing its abundant uploading into EVs. Upon i.m. injection of DNA vectors expressing Nef^mut^-derivatives, nanovesicles containing antigens fused with Nef^mut^ are released by muscle cells, can freely circulate into the body, and can be internalized by antigen-presenting cells (APCs). Thereby, EV-associated antigens are cross-presented to prime antigen-specific CD8^+^ T lymphocytes^13^.

We already tested the efficacy of the Nucleocapsid (N)-specific CD8^+^ T cell immunity elicited by EVs engineered in vivo for the incorporation of SARS-CoV-2 N protein^14^ in survival assays carried out on K18-hACE2 transgenic mice^15^. These mice replicate SARS-CoV-2 by virtue of the expression of the human ACE2 receptor expressed under the control of a human cytokeratin-18 promoter which allows viral entry in epithelial cells^16^. However, it was reproducibly shown that the heavy pathogenic effects observed in infected mice essentially rely on viral invasion of central nervous system, while the infection in lung tissues contributes to the overall health decay and weight loss only marginally^17–20^. Nevertheless, SARS-CoV-2 replicates in lungs of K18-hACE2 mice as efficiently as in lungs of infected humans^16^. To evaluate the translation relevance of our vaccine strategy, we were interested in testing the efficacy of the EV-induced N-specific immunity in counteracting the viral replication in lungs. To this end, both CD8^+^ T cell immunity and viral loads in lungs were measured in mice challenged 3 weeks or 3 months after immunization. The results showing a strong viral inhibition in lungs of N-immunized mice pave the way toward the design of second-generation anti-SARS-CoV-2 vaccines which, considering the extremely low variability of N protein, could be effective against emerging variants.

## Results

### Induction of N-specific CD8^+^ T polyfunctional and CD8^+^ T resident memory lymphocytes in the lungs

**Fig. 1:**
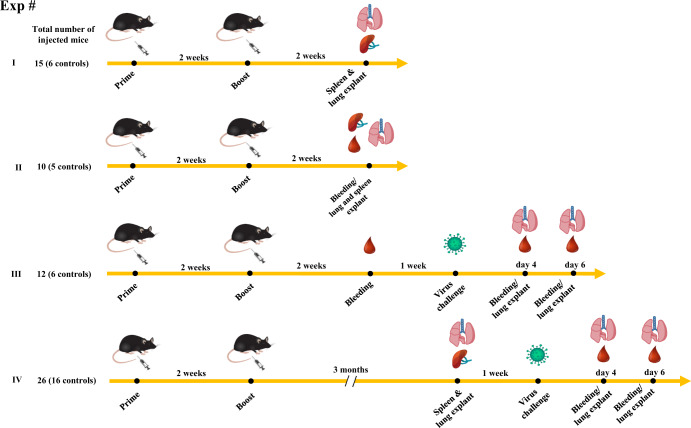
Scheme depicting both flow and time points of the here presented immunization/infection experiments carried out in K18-hACE2 mice. Shown is also the number of animals for each experiment.

**Fig. 2:**
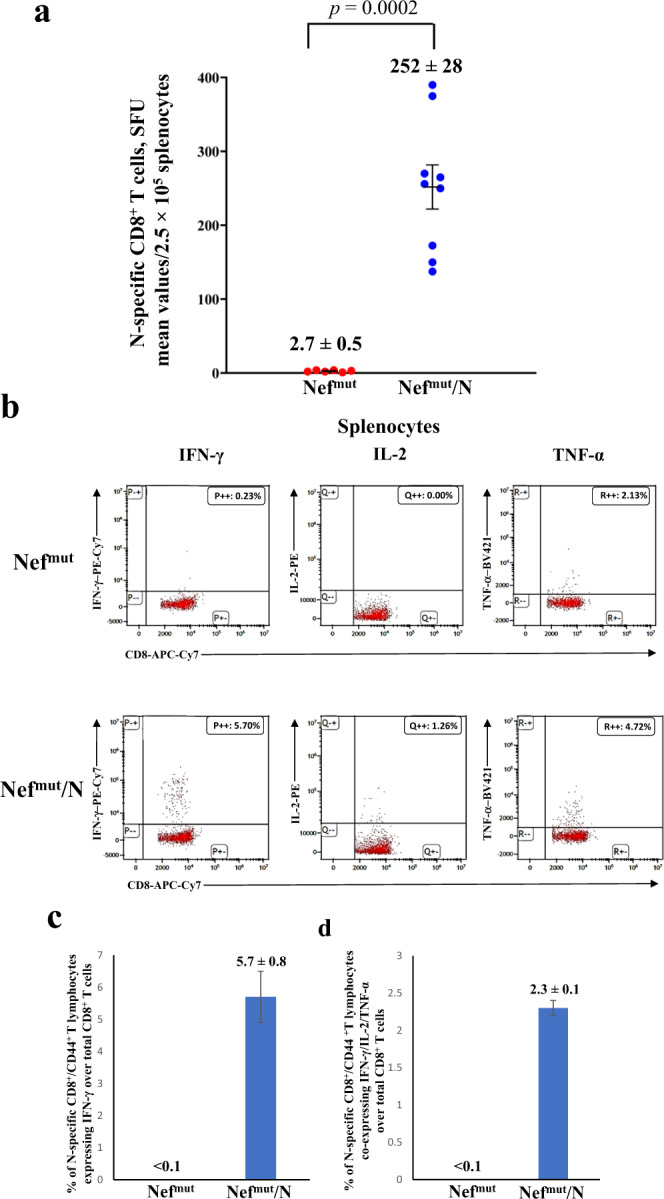
Detection of SARS-CoV-2-N-specific CD8^+^ T cells in splenocytes isolated from K18-hACE2 mice i.m. injected with either Nef^mut^- (6 mice) or Nef^mut^/N- (9 mice) expressing DNA vectors. **a** A total of 2.5 × 10^5^ splenocytes were incubated overnight with 5 μg/ml of either unrelated or SARS-CoV-2-N-specific peptide in IFN-γ EliSpot microwells. Shown are the numbers of spot-forming units (SFUs)/well calculated as mean values of triplicates after subtraction of mean spot numbers detected in wells of splenocytes treated with the unspecific peptide. Reported are intragroup mean values. Two-tailed Mann–Whitney U Test, *p* = 0.0002, error bars, s.e.m. **b**–**d** ICS/flow cytometry analysis of splenocytes isolated from mice injected with vectors expressing either Nef^mut^ or Nef^mut^/N. In panel **b**, raw data from a representative experiment for the detection of IFN-γ, IL-2, and TNF-α are presented. In panel **c**, shown are the percentages of cells expressing IFN-γ over the total of CD8^+^/CD44^+^ T cells from mice injected with the indicated DNA vectors. Shown are mean values of the percentages of IFN-γ expressing cells from at least three pooled cell cultures treated with specific peptides after subtraction of values detected in cultures treated with an unrelated peptide. In panel **d**, percentages of cells simultaneously expressing IFN-γ, IL-2, and TNF-α over the total of CD8^+^/CD44^+^ T cells are presented. Shown are mean values of the absolute percentages of cytokine expressing cells from at least three pooled cell cultures treated with specific peptides after subtraction of values detected in cultures treated with an unrelated peptide. Error bars, s.e.m.

**Fig. 3:**
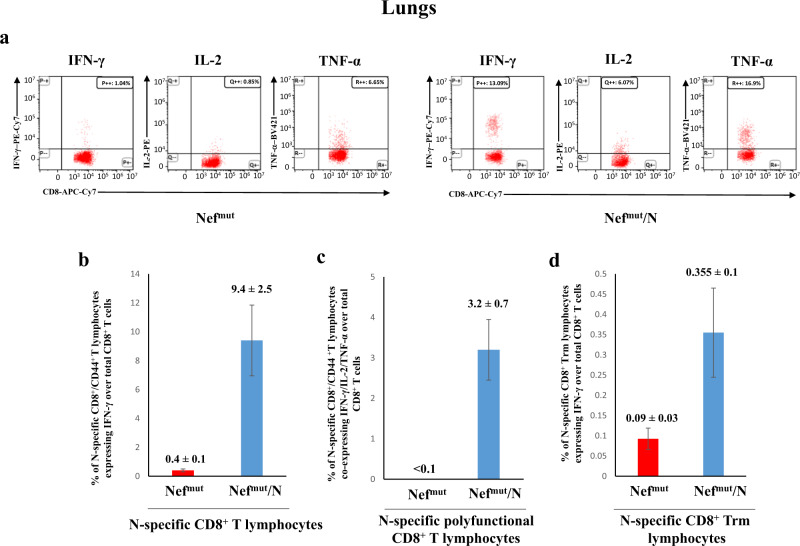
Detection of SARS-CoV-2-N-specific CD8^+^ T lymphocytes in cultures of cells isolated from lungs of K18-hACE2 mice i.m. injected with either Nef^mut^- or Nef^mut^/N- expressing DNA vectors. **a** Raw data from a representative ICS/flow cytometry analysis for the detection of IFN-γ, IL-2, and TNF-α within the CD45^-^ fraction of cells isolated from lungs. **b** Percentages of cells expressing IFN-γ over the total of CD8^+^/CD44^+^ T cells within lung cells pooled from three mice injected with the indicated DNA vectors. Shown are mean values ± SE of percentages of IFN-γ expressing cells from at least three pooled cell cultures treated with specific peptides after subtraction of values detected in cells treated with an unrelated peptide. **c** Percentages of cells simultaneously expressing IFN-γ, IL-2, and TNF-α over the total of CD8^+^/CD44^+^ T cells within lung immune cells pooled from three mice injected with the indicated DNA vectors. Shown are mean values of the percentages ±SE of cytokine expressing cells from pooled cell cultures treated with the N peptide after subtraction of values detected in cultures treated with an unrelated peptide. **d** Percentages of CD8^+^ Trm cells (i.e., CD49a^+^, CD69^+^, CD103^+^) expressing IFN-γ over the total of CD8^+^/CD44^+^ T lymphocytes. Shown are mean value of percentages of positive cells from pooled cell cultures treated with the N peptide after subtraction of values detected in cultures treated with an unrelated peptide. Error bars, s.e.m.

K18-hACE2 transgenic mice were inoculated twice 2 weeks apart with vectors expressing either Nef^mut^ (6 mice) or Nef^mut^/SARS-CoV-2 N fusion protein (9 mice) following the experimental flow depicted in Fig. 1. From 10 to 15 days thereafter, N-specific CD8^+^ T cell immunity was evaluated in both spleen and lungs, in the latter case by exploiting the intravenous injection of a fluorescent anti-CD45 antibody to identify lymphocytes residing in both alveolar and interstitial compartments as the CD45^-^/CD3^+^ cell sub-population^21^. IFN-γ EliSpot analysis for the detection of N-specific CD8^+^ T lymphocytes in spleens was carried out using the N_219–228_ peptide, and revealed a mean of more than 250 spots/2.5 × 10^5^ total splenocytes from vaccinated mice (Fig. 2a, Supplementary Fig. 1), whereas mice injected with Nef^mut^-expressing DNA scored negative. By ICS/flow cytometry analysis, we found that immunization gave rise to a mean of 5.7% of N-specific CD8^+^ T lymphocytes, in the presence of a mean of more than 2% of polyfunctional (i.e., co-expressing IFN-γ, IL-2, and TNF-α), N-specific CD8^+^ T lymphocytes (Fig. 2b–d, Supplementary Fig. 2). When the ICS analysis was carried out in the CD45 negative fraction of cells isolated from lungs (which identified tissue-specific immune cells), higher percentages of both IFN-γ expressing and polyfunctional N-specific CD8^+^ T lymphocytes were found (Fig. 3a–c, Supplementary Fig. 3). In addition, and of a major importance, a well distinguishable population of N-specific T-resident memory (Trm, i.e., CD44^+^, CD49a^+^, CD69^+^, CD103^+^, IFN-γ^+^) CD8^+^ T lymphocytes was found in the lungs of vaccinated mice (Fig. 3d). Notably, a positive correspondence between the percentages of N-specific circulatory and lung CD8^+^ T cells can be found in Nef^mut^/N injected mice (Supplementary Fig. 4). Of note, due to the limited number of recovered cells, cultures from lungs were set by pooling cells from two/three infected mice per group.

We concluded that the immunization with Nef^mut^/N expressing vector induced a strong N-specific CD8^+^ T cell immunity in lung tissues associating with the generation of a sub-population of N-specific CD8^+^ Trm lymphocytes.

### The Nef^mut^ fusion is critical for the induction of N-specific immunity in the lungs

**Fig. 4:**
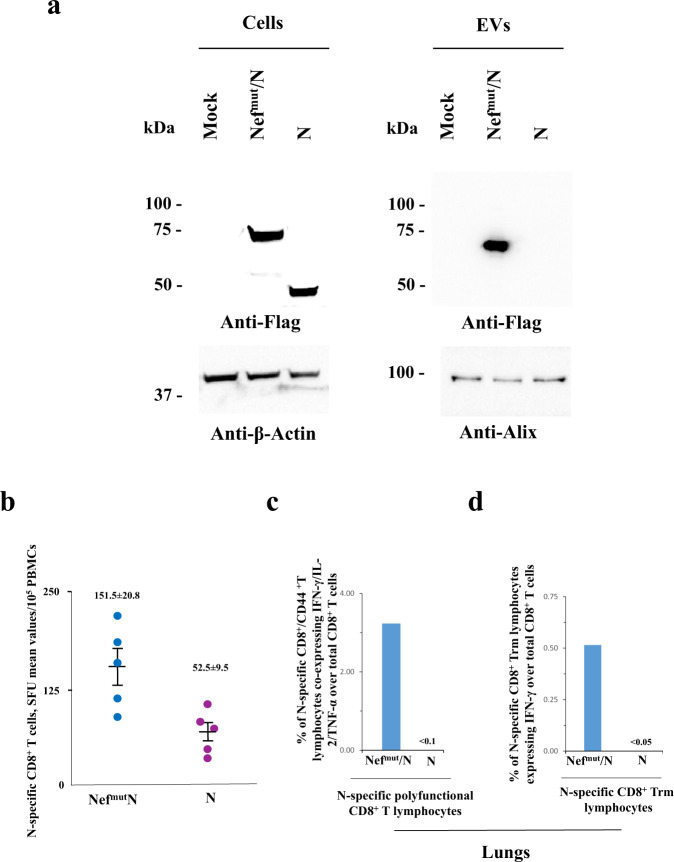
Detection of N-specific CD8^+^ T cells in both blood and lungs of mice injected with N-expressing DNA (*n* = 10). **a** Western blot analysis of 30 μg of lysates from HEK293T cells transfected with DNA vectors expressing either Nef^mut^/N or N. The same assay was carried out on equal volumes of buffer where purified EVs were resuspended after differential centrifugations of respective supernatants. As control, conditions from mock-transfected cells were included. Filters were revealed by anti-flag tag, anti-β-actin, and anti-Alix (i.e., an EV marker) antibodies. Molecular markers are given in kDa. The results are representative of two independent experiments. Blots derived from the same experiment and were processed in parallel. **b** Detection of N-specific CD8^+^ T lymphocytes in PBMCs of K18-hACE2 mice i.m. injected with either Nef^mut^/N- or N-expressing DNA vectors (5 mice per group). Shown are results from IFN-γ EliSpot analysis carried out on 1 × 10^5^ PBMCs incubated overnight with 5 μg/ml of either unrelated or N-specific peptides. Intragroup mean values are indicated, error bars, s.e.m. **c** Percentages of cells simultaneously expressing IFN-γ, IL-2, and TNF-α over the total of CD8^+^/CD44^+^ T cells isolated from lungs of mice injected with the indicated DNA vectors. **d** Percentages of lung CD8^+^ Trm cells (i.e., CD49a^+^, CD69^+^, CD103^+^) expressing IFN-γ over the total of lung CD8^+^/CD44^+^ T lymphocytes from mice injected with the indicated DNA vectors. For both **c** and **d** panels, shown are mean values of percentages of positive cells from pooled cell cultures treated with specific peptides after subtraction of values detected in cultures treated with an unrelated peptide.

We previously reported that the fusion of Nef^mut^ with either HPV16-E6 or -E7 viral proteins was critical for the induction of an effective antigen-specific CD8^+^ T cell immunity in circulating cells^22^. To test whether the Nef^mut^ fusion is necessary for the generation of N-specific immunity in the lungs as well, we immunized K18-hACE2 transgenic mice by injecting DNA vectors expressing either N alone or Nef^mut^/N following the above reported prime-boost design. Results from preliminary western blot experiments carried out on EVs isolated from supernatants of transiently transfected 293T cells showed that, as expected, the N protein did not associate with EVs when expressed alone (Fig. 4a and Supplementary Fig. 5).

Fifteen days after the second immunization, immune cells from both blood and lungs of injected mice were isolated. The results from the analysis of both polyfunctional and resident memory N-specific CD8^+^ T lymphocytes in the lungs showed the lack of N-specific cells in mice injected with the DNA vector expressing N alone, in the presence of the induction of a limited number of N-specific, circulatory CD8^+^ T lymphocytes. Conversely, and as expected, the CD8^+^ T cell-specific immune response was readily detectable in cells from lungs of mice injected with the Nef^mut^/N-expressing DNA, in the presence of a higher number of N-specific circulatory CD8^+^ T lymphocytes (Fig. 4b–d).

These data support the idea that the generation of engineered EVs dictated by the Nef^mut^-fusion was a key step for the induction of N-specific CD8^+^ T cell immunity in the lungs.

### Impaired SARS-CoV-2 replication in lungs of Nef^mut^/N-immunized mice

**Fig. 5:**
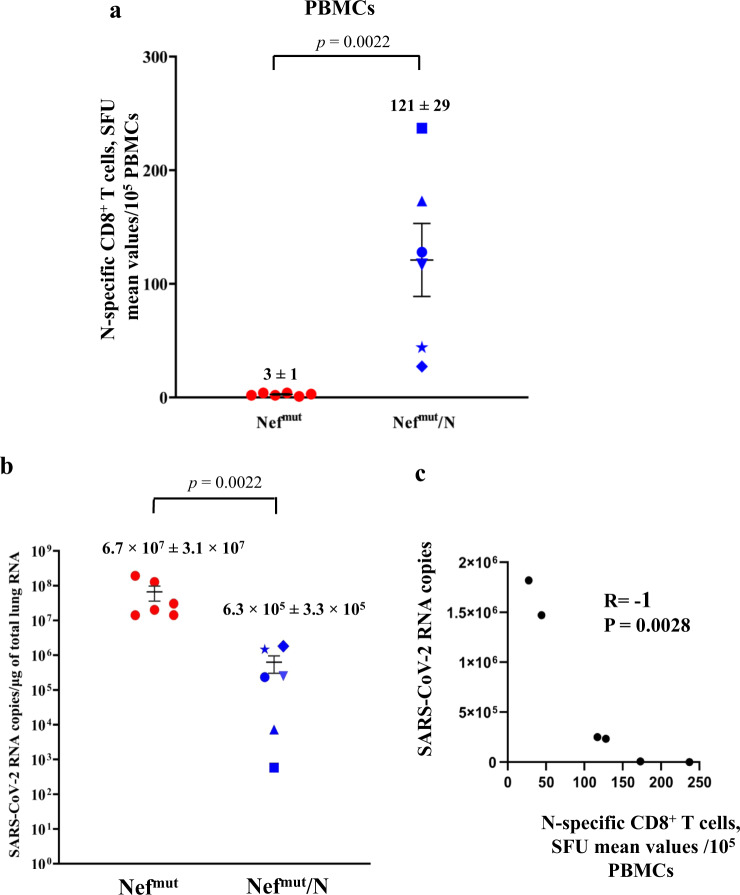
Viral loads in lungs of immunized mice. **a** Detection of N-specific immunity in vaccinated mice (6 mice per group). A total of 10^5^ PBMCs were incubated overnight with or without 5 μg/ml of either unrelated or SARS-CoV-2-N-specific peptides in IFN-γ EliSpot microwells. Shown are the numbers of SFUs/well calculated as mean values of triplicates after subtraction of mean spot numbers detected in wells of PBMCs treated with an unspecific peptide. Reported is intragroup mean value. Two-tailed Mann–Whitney U Test *p* < 0.05, error bars, s.e.m. **b** Viral loads in lungs of immunized/infected mice. Four and six days after challenges, injected mice (6 per group) were sacrificed, and lungs processed for the extraction of total RNA. One μg of total RNA from each infected mouse was then analyzed by RT-qPCR for the presence of SARS-CoV-2 N-specific RNAs. As internal control, actin RNA was also amplified. Shown are the N viral RNA copies amplified from total RNA isolated from lungs of each animal, together with intragroup mean values. two-tailed Mann–Whitney U Test *p*: 0.0022, error bars, s.e.m. **c** Graph of the two-tailed Spearman Rank Correlation Test showing the inverse correlation between pre-existing N-specific CD8^+^ T cells and viral loads in lungs of immunized/infected mice.

The effectiveness of any antiviral vaccine strategy should be evaluated in terms of its efficacy in reducing viral replication in the target tissue. In the case of SARS-CoV-2 infection, the assessment should be focused on the lungs, considering that in humans Covid-19-related severe disease and death are correlated with an uncontrolled viral replication in pulmonary alveoli. To this aim, in a third immunization experiment, mice (6 per group) were injected with either Nef^mut^ or Nef^mut^/N DNA vectors, and actual immunization was checked fifteen days after the second immunization by IFN-γ EliSpot assay on peripheral blood mononuclear cells (PBMCs, Fig. 5a). After additional 7 days, mice were infected intranasally (i.n.) with 4.4 lethal doses of SARS-CoV-2, and the lungs were isolated 4–6 days thereafter. Lung tissues were then processed for the extraction and purification of total RNA. RT-qPCR analysis was finally carried out to enumerate the copy numbers of SARS-CoV-2 N-related RNA molecules. Overall, we found that virus replicated in lungs of N-vaccinated mice more than 60-fold less efficiently than in control mice (Fig. 5b; two-tailed Mann–Whitney U Test, *p* = 0.0022). Consistently with what previously reported in survival assays^15^, the inhibition of virus replication appeared most effective (i.e., more than 3.5 logs of reduction) in mice that had developed stronger N-specific immune responses. In line with this observation, two-tailed Spearman Rank Correlation Test showed a negative correlation between pre-challenge N-specific IFN-γ EliSpot and days 4 and 6-cumulative results of RT-qPCR assays on N viral gene (Spearman R = −1, *p* = 0.0028) (Fig. 5c).

### Intranasal SARS-CoV-2 infection does not affect the pre-existing N-specific cell immunity in lungs

**Fig. 6:**
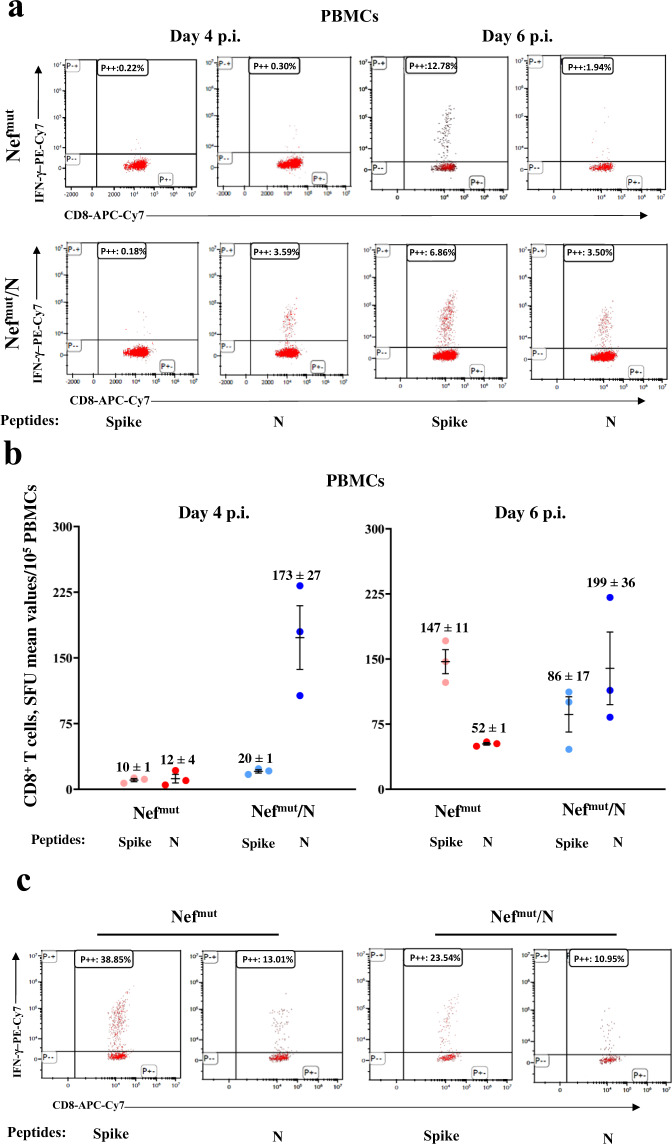
Detection of SARS-CoV-2-N-specific CD8^+^ T cells in K18-hACE2 mice i.m. injected with either Nef^mut^- or Nef^mut^/N-expressing DNA vectors and then infected. **a** Representative results from ICS/flow cytometry analysis for the expression of IFN-γ in PBMCs from mice injected with the indicated DNA vectors and then infected. Cells harvested 4 and 6 days after virus infection were cultivated overnight with either Spike-, N-specific, or unrelated peptides. Shown are data obtained with PBMCs pooled from three infected mice per condition representative of two experiments. Quadrants were set on the basis of cell fluorescence of samples treated with the unrelated peptide. **b** Detection by IFN-γ EliSpot analysis of both Spike- and N-specific CD8^+^ T cells within PBMCs from vaccinated mice both 4 and 6 days after infection. A total of 10^5^ PBMCs were incubated overnight with or without 5 μg/ml of either unrelated, Spike- or N- specific peptides in IFN-γ EliSpot microwells. Shown are the numbers of SFUs/well calculated as mean values of triplicates after subtraction of mean spot numbers found in wells of splenocytes treated with an unspecific peptide. Reported are intragroup mean values, error bars, s.e.m. **c** ICS/flow cytometry analysis for the detection of IFN-γ expressing CD8^+^ T lymphocytes within immune cells isolated 6 days after infection from lungs of K18-hACE2 mice injected with either Nef^mut^- or Nef^mut^/N-expressing DNA vectors. Cells pooled from lungs of three mice were incubated overnight with either Spike-, N-specific or unrelated peptides. Results are representative of two experiments. Quadrants were set as for panel **a**.

It is well known that SARS-CoV-2 replication in lungs couples with hyper-inflammation and profound immune dysregulation^23,24^. Hence, we next were interested in monitoring whether and to what extent SARS-CoV-2 infection influenced the state of SARS-CoV-2-N-specific immunity in both circulating cells and lungs of vaccinated mice. To this aim, PBMCs recovered from infected mice were analyzed at both 4 and 6 days after challenge by IFN-γ EliSpot and ICS/flow cytometry assays using either N_219–228_ or Spike_539–546_ peptides (Fig. 6a, b). We noticed that at day 6 post-infection the Spike-specific CD8^+^ T cell immunity jumped dramatically in control mice, whereas it increased at apparently lower extents in N-vaccinated ones in the presence of a persistent N-specific immunity. Analysis on cumulative results from day 4- and day 6-N-specific IFN-γ EliSpot assay showed a statistically significant higher N-specific response in Nef^mut^/N vaccinated mice compared to control animals (two-tailed Student T Test with Welch correction, *p* = 0.0015). When the ICS/flow cytometry analysis was carried out in cells isolated from the lungs of mice 6 days after challenge, high levels of both Spike- and N-specific CD8^+^ T cell immunity were found in control mice that conversely appeared lower in Nef^mut^/N-immunized group (Fig. 6c).

These data indicated that the infection did not relevantly affect the levels of N-specific immunity in lungs of N-vaccinated mice.

### Persistent N-specific CD8^+^ T cell immunity in lungs of vaccinated mice

**Fig. 7:**
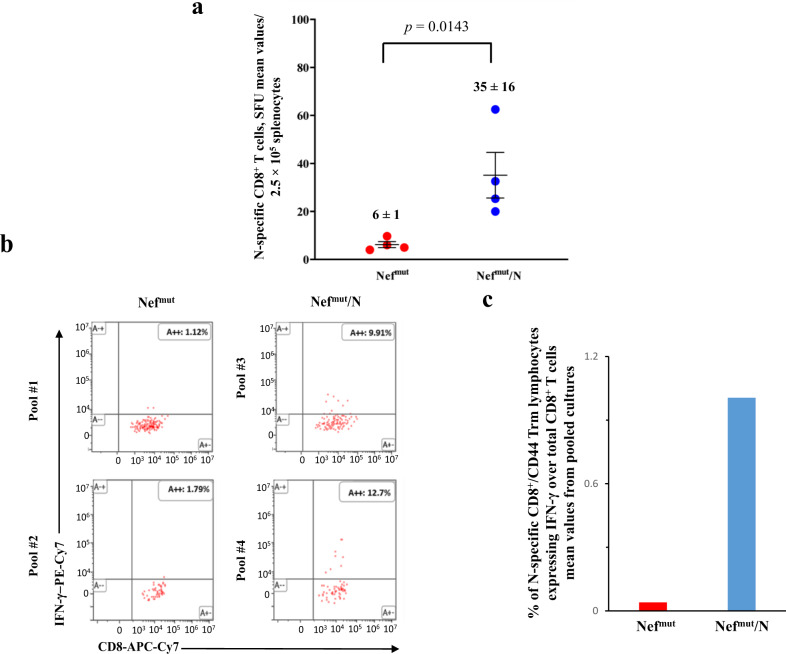
Detection of SARS-CoV-2-N-specific CD8^+^ T lymphocytes in K18-hACE2 mice three months after i.m. injection with either Nef^mut^- or Nef^mut^/N- expressing DNA vectors (4 per group). **a** Detection of N-specific CD8^+^ T cells in spleens of injected mice. A total of 2.5 × 10^5^ splenocytes were incubated overnight with or without 5 μg/ml of either unrelated or SARS-CoV-2-N-specific peptides in IFN-γ EliSpot microwells. Shown are the numbers of SFUs/well calculated as mean values of triplicates after subtraction of mean spot numbers calculated in wells of splenocytes treated with an unspecific peptide. Reported are intragroup mean value. One-tailed Mann–Whitney U Test, *p* = 0.0143, error bars, s.e.m. **b** Raw data from two ICS/flow cytometry analysis for the detection of IFN-γ expressing CD8^+^ Trm lymphocytes within immune cells isolated from lungs of K18-hACE2 mice injected with either Nef^mut^- or Nef^mut^/N-expressing DNA vectors. The lungs of immunized mice were explanted after intravenous injection of fluorescently labeled anti-CD45 mAb. Then, pools each formed by the cells extracted from the lungs of two mice injected with either Nef^mut^- (pools # 1 and 2) or Nef^mut^/N- (pools # 3 and 4) DNA vectors were cultivated in the presence of either N-specific or unspecific peptides. Reported are the percentages of IFN-γ expressing cells over the total of the CD8^+^ Trm sub-populations. **c** Percentages of CD8^+^ Trm cells expressing IFN-γ over the total of CD8^+^/CD44^+^ T lymphocytes isolated from lungs of mice injected with the indicated DNA vectors. Shown are mean values of percentages of positive cells from cultures treated with specific peptides after subtraction of values detected in cells treated with an unrelated peptide. The results are from two cell cultures each formed by the pool of cells from two immunized mice.

The persistence of immune protection over time is a mandatory feature for any vaccine. In the case of EV-induced N-specific CD8^+^ T cell immunity, the prompt generation of an antigen-specific CD8^+^ Trm sub-population in lungs was suggestive of a durable immune response. To test this hypothesis, the N-specific immune response was monitored three months after the second shot in a fourth immunization experiment. IFN-γ EliSpot analysis on splenocytes isolated from four mice per group showed the persistence of N-specific circulatory CD8^+^ T lymphocytes (Fig. 7a), although at reduced levels compared to what observed three weeks after the second injection. The lungs of these mice were explanted after intravenous injection of fluorescently labeled anti-CD45 mAb. Then, the immune cells were isolated, and cultures each formed by pooling the cells from two mice were run in the presence of either N-specific or unspecific peptides. Authentically lung-associated cells were identified by flow cytometry analysis as the CD45 unlabeled cell fraction. Notably, N-specific lung Trm scored more than 9% of total lung CD8^+^ Trm in mice injected with the Nef^mut^/N vector (Fig. 7b). In addition, the percentages over the total of lung CD8^+^ T lymphocytes (Fig. 7c) apparently increased compared to those observed 2 weeks after boosters. These results were in line with the hypothesis that the injection of Nef^mut^/N DNA vector generated a long-lasting immunity in lungs.

### Antiviral activity in lungs three months after immunization

**Fig. 8:**
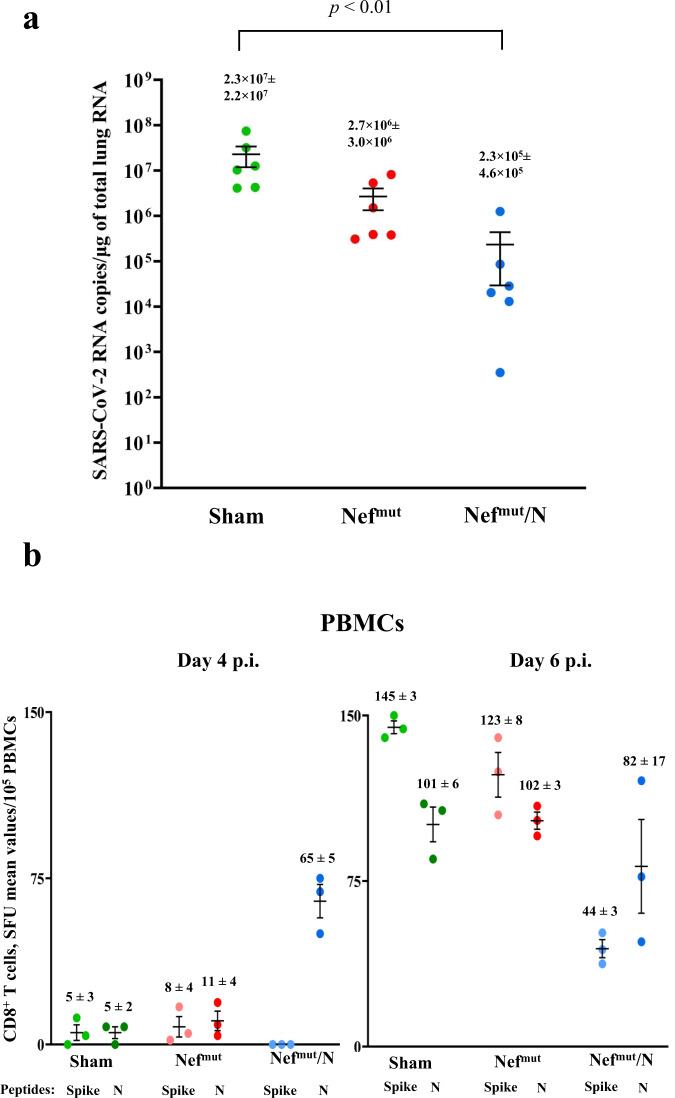
Viral loads in lungs of mice infected 3 months after last injections. **a** RT-qPCR analysis. Four to six days after challenge, injected mice (6 per group) were sacrificed, and lungs processed for the extraction of total RNA. One μg of total RNA from each infected mouse was then analyzed by RT-qPCR for the presence of SARS-CoV-2 N-specific RNAs. As internal control, actin RNA was also amplified. Shown are the N-specific viral RNA copies amplified from total RNA isolated from lungs of each animal, together with intragroup mean values. *p < 0.01, error bars, s.e.m. **b** Detection of both Spike- and N-specific CD8^+^ T cells within PBMCs isolated both 4 and 6 days after infection (3 mice per group). A total of 10^5^ PBMCs were incubated overnight with or without 5 μg/ml of either unrelated, Spike- or N- specific peptides in IFN-γ EliSpot microwells. Shown are numbers of SFUs/well calculated as mean values of triplicates after subtraction of mean spot numbers calculated in wells of splenocytes treated with unspecific peptides. Reported are intragroup mean values, error bars, s.e.m.

Finally, we were interested to establish whether the persistence of N-specific Trm in lungs of vaccinated mice associated with an antiviral state. Six mice from the last immunization experiment were infected i.n. with the identical experimental conditions used in the previous challenges. In view of the already described activation effect of Nef^mut^ on immature dendritic cells^25^, which would favor the natural antiviral immune response, a group of six mice injected with saline solution (sham) was included in the challenge experiment. The RT-qPCR analysis of total RNA isolated from lungs 4–6 days after challenge showed an overall drop of virus replication extent in N-immunized mice compared to controls (Fig. 8a) similarly to what observed upon challenge three weeks after boost. Kruskal–Wallis Test followed by Dunn’s Multiple Comparisons Test showed a statistically significant control of infection in Nef^mut^/N-vaccinated mice compared to the sham group in terms of mean of N-related viral gene copies, with *p* < 0.01. Interestingly, the immune response induced by Nef^mut^ alone seemed to contribute to the total antiviral effect induced by the immunization against Nef^mut^/N. The infection generated a potent Spike-related CD8^+^ T cell immune response in control mice, as assessed by IFN-γ EliSpot analysis carried out on PBMCs isolated at both 4 and 6 days after challenge. Differently, the Spike-specific CD8^+^ T cell immune response in N-immunized mice was elicited at lower levels (Kruskal–Wallis Test followed by Dunn’s Multiple Comparisons Test, Nef^mut^/N-vaccinees vs sham mice, at day 6: *p* < 0.05), in the presence of an N-specific immunity not diminished compared to pre-infection levels (Fig. 8b).

Taken together, these data support the idea that CD8^+^ T N-specific immunity induced in lungs can control virus replication three months after boosting.

## Discussion

Respiratory airways are the battlefield where the fate of persons infected by SARS-CoV-2 is determined. The efficacy of anti-SARS-CoV-2 vaccines would be the result of promptness, potency, and durability of the immune response evoked in both upper and lower respiratory tracts. In humans, current mRNA-based vaccines have been shown to induce immune responses quantitatively limited and qualitatively incomplete in both tracts^26^. Second-generation vaccines are expected to overcome such limitations by focusing their immunogenic activity at pulmonary level. In this perspective, we investigated the potentialities of an innovative anti-SARS-CoV-2 CD8^+^ T cell-based vaccine consisting in engineered EVs spontaneously released by muscle cells incorporating the SARS-CoV-2 N protein. They are produced upon i.m. injection of a DNA vector expressing the SARS-CoV-2 N protein fused at the C-terminus of Nef^mut^, i.e., an EV-anchoring protein incorporating in EVs at very high efficiency even when fused with heterologous proteins^27^. Of importance, we previously demonstrated that Nef^mut^-dependent antigen incorporation into EVs is critical for the induction of circulatory antigen-specific CD8^+^ T lymphocytes, which were induced much less efficiently when the antigen was expressed alone^22^. The here presented data extend this finding to the antigen-specific CD8^+^ T cell immunity induced in lungs, i.e., a distal tissue district respect to the injection site.

This model has been previously investigated in survival assays using K18-hACE2 transgenic mice and SARS-CoV-2 N engineered EVs as immunogen^15^. However, such an animal model recapitulates the Covid-19 pathogenesis occurring in humans only partly. In fact, results from different papers demonstrated that, upon i.n. challenge, the most evident clinical signs of infection derive almost exclusively from the viral neuroinvasion, with cells of olfactory bulbs acting as viral port of entry^17–20^. Hence, evaluating the efficacy of anti-SARS-CoV-2 compounds in K18-hACE2 transgenic mice by measuring weight loss, ataxia, and death could lead to results of not obvious significance for humans. Conversely, since SARS-CoV-2 replicates efficiently in human lungs, monitoring the antiviral effects in terms of virus replication in lungs of transgenic mice would be much more informative for a possible translation in humans.

Using fluorescent EVs, several authors demonstrated that circulating EVs diffuse in lungs quite efficiently^28–31^. We assume that engineered EVs emerging from muscle cells expressing Nef^mut^/N fusion products freely circulate into the body reaching mediastinal lymph nodes and lung germinal centers, where N-specific CD8^+^ T lymphocytes are activated, leading to the generation of N-specific CD8^+^ Trm also. This mechanism might explain how a distal i.m. injection could result in a sustained cell immunity in lungs.

We measured the viral replication levels in lungs of vaccinated mice through RT-qPCR analysis.

Several authors reported that the i.n. infection of K18-hACE2 transgenic mice leads to a peak of virus replication in the lungs after 2–3 days^17,19,32–34^. The extents of viral replication then decrease, remaining stable until days 7 post challenge^33,34^. These evidences justified the gathering of data we obtained with lungs excised at days 4 and 6 after infection.

In the evaluation of these data, it should be considered that, despite the high sensitivity of the assay, it could not distinguish RNA viral molecules incorporated into infectious viral particles from those associated with non-infectious ones. On the other hand, the assay cannot discriminate cell-associated viral RNA molecules which will be allocated in emerging viral particles. In addition, one should consider the antiviral mechanism on the basis of the CD8^+^ T cell immunity. In fact, differently from neutralizing antibodies which prevent the entry of virus into target cells, virus-specific CD8^+^ T lymphocytes can control viral replication by recognizing and destroying cells exposing virus-related peptides loaded on MHC Class I, when high levels of intracellular viral RNA have been already produced. Finally, besides controlling the virus spread, the CD8^+^ T cell-mediated killing of infected cells would be beneficial also in terms of the generation of an environment facilitating the antiviral action of the natural immune response.

RT-qPCR data obtained in mice challenged 3 months after immunization suggested that the response to Nef^mut^ alone might be part of the total antiviral effect observed in mice injected with Nef^mut^/N.

In view of the previously documented activation effect of Nef^mut^ on dendritic cells (DCs)^25^, it is tempting to speculate that the total, i.e., natural plus vaccine-induced, N-specific CD8^+^ T cell immune response would be favored by a sort of persistent pre-activation state (i.e., trained immunity) of pulmonary dendritic cells induced by Nef^mut^ after internalization of engineered EVs. Notably, in light of these considerations based on the results obtained three months after boosting, it cannot be excluded that the Nef^mut^/N-induced antiviral effect observed 3 weeks after last immunizations was underestimated.

We noticed that the levels of emerging Spike-specific CD8^+^ T lymphocytes in lungs appeared reduced in vaccinated mice compared to control ones. We assumed that this effect was consequence of the inhibitory effect on viral replication. On the other hand, the detection of a quantitatively similar N-specific sub-population of CD8^+^ Trm lymphocytes both 3 weeks and 3 months after boosting was strongly suggestive of the establishment of a long-lasting anti-SARS-CoV-2 immunity at lungs. The strong reduction of viral replication extents we detected 3 months after vaccination was in line with this idea. Considering that it was largely demonstrated that lung CD8^+^ Trm can duplicate even in absence of antigenic stimulus^35^, it is conceivable that this antiviral immunity has the potential to persist for a very long time. Based on here presented experimental evidences, lung N-specific CD8^+^ Trm might be considered the most appropriate immunologic correlate of the observed inhibition of viral replication in lungs.

Our study presents a number of limitations. First, virus loads from lungs of challenged mice were not evaluated in terms of infectious units. However, a strict correlation between viral titers and number of RNA copies in lungs of SARS-CoV-2-infected K18-hACE2 mice has been previously demonstrated^17^, and in many instances viral loads were measured exclusively in terms of number of RNA copies, as in the assessment report for the efficacy evaluation of the Spikevax vaccine in K18-hACE2 mice^36^. Additional limitations are as follows: the antiviral effect in lungs of vaccinated mice was evaluated using a single, very high viral dose, i.e., 4.4. LD_50_; dose-response experiments with different amounts of injected DNA are lacking; the contribution to the overall antiviral effect of an N-specific CD4^+^ T lymphocyte response in lungs cannot be excluded; and no data concerning the phenotypic cell signature of Nef^mut^/N-induced CD8^+^ T lymphocytes are available. On this subject, due to the several peculiarities of the Nef^mut^-based vaccine platform, e.g., the need of EVs for antigen delivery, the absence of adjuvants, and the intrinsic ability of Nef^mut^ to activate DCs^25^, one may speculate that the CD8^+^ T cell signature would be unique and differing, for instance, from that recently described for a peptide-based CD8^+^ T cell-specific anti-SARS-CoV-2 vaccine^37^.

Due to its quite limited variability, N protein represents a privileged immunologic target to fight emerging SARS-CoV-2 variants. Notably, it was reported that N-specific T cells showed cross-reactivity among different endemic human Coronaviruses, and their numbers correlated with protection against severe Covid-19 disease^38^. In addition, the number of both N-specific CD4^+^ and CD8^+^ T circulating lymphocytes was shown to inversely correlate with inflammation and viral load in upper airways^39^.

Intranasal vaccines would represent a complementation/amelioration of current vaccines, which are not effective enough to impede virus replication in respiratory mucosa^40–44^. On this subject, the intranasal administration in mice of an anti-SARS-CoV, CD4^+^ T cell-specific vaccine was found more effective than its i.m. injection^45^. More recently, an intranasal Sendai virus vector-derived anti-SARS-CoV-2 vaccine was shown to induce a powerful antiviral CD8^+^ T cell immunity^46^. The presented results, together with those very recently published regarding the immunogenicity of SARS-CoV-2 N-engineered EVs in ex vivo human cells^47^, can be instrumental to the design of a second-generation, innovative anti-Covid-19 vaccine based on mucosal administration of N-engineered EVs. In this way, optimal levels of CD8^+^ T cell immunity should be achieved at the viral port of entry.

## Methods

### DNA constructs

Open-reading frames coding for either Nef^mut^ alone or fused with SARS-CoV-2 N protein were cloned into pVAX1 DNA vector (Thermo Fisher, Waltham, MA). To obtain the pVAX1 vector expressing Nef^mut^, its open reading frame was cloned into Nhe I and EcoR I sites. To recover the vector expressing Nef^mut^-N, an intermediate vector referred to as pVAX1/Nef^mut^fusion was produced. Here, the whole Nef^mut^ ORF deprived of its stop codon was followed by a sequence coding a GPGP linker including a unique Apa I restriction site. In this way, N sequences comprising the Apa I site at their 5’ end, and the Pme I one at their 3’ end were fused in frame with Nef^mut^ open reading frame. Stop codons of SARS-CoV-2-related sequences were preceded by sequences coding for a DYKDDDK epitope tag (flag-tag). In the Nef^mut^/N construct, the entire open reading frame for N protein (422 aa), except the M_1_ amino acid, was part of the fusion product. To express the SARS-CoV-2 N protein, the entire sequence from the SARS-CoV-2/Italy INMI1#52284 (SARS-Related Coronavirus 2, isolate Italy-INMI1, NR-52284, deposited by Dr. Maria R. Capobianchi for distribution through BEI Resources, NIAID, and NIH) was cloned into the pVAX1 vector, with a flag-tag inserted just before the stop codon. SARS-CoV-2 sequences were optimized for expression in human cells through GenSmart™ Codon Optimization software from Genescript (Piscataway, NJ). All vectors were synthesized by OfficinaeBio (Venice, Italy).

### Animals and authorizations

Six-week-old, female C57 Bl/6 K18-hACE2 transgenic mice were purchased from Charles River (Calco, Italy) and hosted at the Central and BSL3 Animal Facilities of the Istituto Superiore di Sanità, as approved by the Italian Ministry of Health, authorizations 565/2020 and 591/2021, and released on 3 June 2020 and 30 July 2021, respectively. Before the first procedure, Datamars (Lugano, Switzerland) microchips were inserted subcutaneously on the dorsal midline between the shoulder blades.

### Mice immunization

Isoflurane-anesthetized mice were inoculated i.m. with 10 μg of DNA in 30 μL of sterile, 0.9% saline solution. DNA injection was immediately followed by electroporation at the site of inoculation with an Agilpulse BTX (Holliston, MA) device, using a 4-needle electrode array (4 mm gap, 5 mm needle length), and applying the following parameters: 1 pulse of 450 V for 50 μs; 0.2 ms interval; 1 pulse of 450 V for 50 μs; 50 ms interval; 8 pulses of 110 V for 10 ms with 20 ms intervals. Mice were immunized into both quadriceps, twice, 2 weeks apart. Mice were sacrificed by cervical dislocation.

### IFN-γ EliSpot analysis

Either 2.5 (for splenocytes) or 1 (for peripheral blood mononuclear cells, PBMCs) ×10^5^ live cells were seeded in triplicate in microwells of 96-multiwell plates (Millipore, Burlington, MA) previously coated with 1:100 diluted anti-mouse IFN- AN18 mAb (Mabtech, Nacka Strand, Sweden, cat. 3321-3-250) in RPMI 1640, 10% heat-inactivated fetal calf serum (FCS, Gibco, Thermo Fisher), and 50 μM 2-mercaptoethanol. Cell cultures were carried out for 16 h in the presence of 5 μg/mL of CD8-specific H2-b-binding SARS-CoV-2 Spike: 539–546 VNFNFNGL^48^, N: 219–228 ALALLLLDRL^49^, or Nef: 48–56 TAATNADCA^50^ peptide. As negative controls, 5 μg/mL of unrelated H2-b binding peptides were used. Peptide preparations were obtained from BEI resources. To check for cell responsiveness, 10 ng/mL phorbol 12-myristate 13-acetate (PMA, Sigma, St. Louis, MO) plus 500 ng/mL of ionomycin (Sigma) were added to the cultures. After 16 h, the cells were discarded, and the plate was incubated for 2 h at room temperature with 1:1000 diluted R4-6A2 biotinylated anti-IFN-γ antibody (Mabtech, cat. 3321-6-250) at the concentration of 100 μg/mL. Wells were then washed and treated for 1 h at room temperature with 1:1000 diluted streptavidin-ALP from Mabtech. Afterward, 100 μL/well of SigmaFast BCIP/NBT were added to the wells to develop spots. Spot-forming cells were finally analyzed and counted using an AELVIS EliSpot reader (Hannover, Germany).

### Cell isolation from spleens, blood, and lungs

PBMCs were recovered from EDTA-blood samples obtained through retro-orbital puncture under topical anesthesia. Erythrocytes were removed by treatment with ACK lysing buffer (Gibco) according to the manufacturer’s instructions.

To isolate splenocytes, spleens were explanted, placed into tubes containing 1 mL of RPMI 1640 and 50 µM 2-mercaptoethanol, then transferred into 60 mm Petri dishes with 2 mL of the same medium. Splenocytes were obtained by notching the spleen sac and pushing the cells out with the plunger seal of a 1 mL syringe. After addition of 2 mL of medium, cells were transferred into a 15 mL conical tube, and the Petri plate washed with 4 mL of medium to maximize cell recovery. Afterward, cells were collected by centrifugation, resuspended in RPMI complete medium containing 50 µM 2-mercaptoethanol and 10% FCS, and counted.

For lung cell isolation, circulating blood cells were fluorescently labeled with 2 μg (10 µl of the commercial stock) of an anti-CD45 antibody (Tonbo-Bioscience, S. Diego, CA anti-mouse CD45.2-APC, cat. 20-04540U100) diluted in 200 µl of 1×PBS inoculated in the tail vein exactly 3 min before cervical dislocation. For cell recovery, lungs were excised, washed with 1×PBS, cut into small pieces, and then digested for 30 min under gentle agitation at 37 °C with 7 mL of 4 mg/mL of type III collagenase (Worthington Biochemical, Lakewood, NJ) and 0.05 mg/mL of DNase I (Sigma) in 1×PBS. After digestion, an equal volume of medium was added and cells were passed through a 70 µm cell strainer, washed, and resuspended in 1×PBS/ACK 1:1 for red blood cells lysis. Finally, the isolated cells were resuspended in complete culture medium and counted.

### Intracellular cytokine staining (ICS) and flow cytometry analysis

Cells collected from blood, spleens, and lungs were cultured at 1 × 10^7^/mL in RPMI medium, 10% FCS, 50 µM 2-mercaptoethanol (Sigma), 1 µg/mL brefeldin A (BD Biosciences, Franklin Lakes, NJ), and in the presence of 5 μg/mL of either Spike, N, or unrelated H2-b CD8^+^ T-specific peptides. Positive controls were conducted by adding 10 ng/mL PMA (Sigma) plus 1 µg/mL ionomycin (Sigma). After 16 h, cells were stained with 1 µL of LIVE/DEAD Fixable FVD-eFluor506 Dead Cell reagent (Invitrogen Thermo Fisher) in 1 mL of 1×PBS for 30 min at 4 °C, and excess dye removed by 2 washes with 500 µL of 1×PBS. Non-specific staining was minimized by pre-incubating cells with 0.5 µg of Fc blocking mAbs (i.e., anti-CD16/CD32 antibodies, Invitrogen/eBioscience Thermo Fisher, cat. 12-7021-82) in 100 µL of 1×PBS with 2% FCS for 15 min at 4 °C. Staining for cell surface markers was performed upon incubation for 1 h at 4 °C with 2 µL of the following anti-mouse Abs: FITC-conjugated anti-CD3 (clone 17A2, cat. 555274, BD Biosciences), APC-Cy7-conjugated anti-CD8a (clone53-6.7, cat. 557654, BD Biosciences), PerCP-conjugated anti-CD4 (clone RM4-5, cat. 553052, BD Biosciences), and BUV395-conjugated anti-CD44 (clone IM7, cat. 740215, BD Biosciences). CD8^+^ T-resident memory (Trm) cells were identified by staining with BUV750-conjugated anti-CD49a (clone Ha31/8, cat. 746854, BD Biosciences), PECF594-conjugated anti-CD69 (clone H1.2F3, cat. 562455, BD Biosciences), and BUV563-conjugated anti-CD103 (clone M290, cat. 741261, BD Biosciences). For intracellular cytokine staining (ICS), cells were fixed and permeabilized using the Cytofix/Cytoperm kit (BD Biosciences), according to the manufacturer’s recommendations. Thereafter, cells were labeled for 1 h at 4 °C with 2 µL of the following Abs: PE-Cy7-conjugated anti-IFN-γ (eBioscience, Thermo Fisher, clone XMG1.2, cat. 25-7311-82), PE-conjugated anti-IL-2 (Invitrogen/eBioscience Thermo Fisher, clone JES6-5H4, cat. 12-7021-82), and BV421 rat anti-TNF-α (BD Biosciences, clone MP6-XT22, cat. 563387) in a total of 100 µL of 1× Perm/Wash Buffer (BD Biosciences). After two washes, cells were fixed in 200 µL of 1× PBS/formaldehyde (2% v/v). Samples were then acquired by a CyotFLEX LX (Beckman Coulter, Brea, CA, USA) flow cytometer and analyzed using Kaluza software (Beckman Coulter). Gating strategy was as follows (Supplementary Figs. 2 and 3): live cells as assessed by LIVE/DEAD dye vs. FSC-A, singlet cells from FSC-A vs. FSC-H (singlet 1) and SSC-A vs. SSC-W (singlet 2), CD3^+^ cells from CD3-FITC vs. SSC-A, CD8^+^, or CD4^+^ cells from CD8-APC-Cy7 vs. CD4-PerCP. CD3^+^/CD8^+^ cell population was gated against CD44^+^ cells, and, to detect polyfunctional CD8^+^ T lymphocytes, the population of cells positive for both CD8 and CD44 was analyzed for APC-Cy7, PE, and BV421 to detect simultaneous changes in IFN-γ, IL-2, and TNF-α production, respectively. To detect CD8^+^ T resident memory (Trm), the population of cells positive for both CD8 and CD44 was analyzed for the co-expression of CD49a, CD69, and CD103. Boolean gates were created to measure co-expression patterns.

### Western blot analysis

Human embryonic kidney (HEK) 293T cells (ATCC, CRL-11268) were grown in DMEM (Gibco) plus 10% FCS (Gibco). Transfection assays were performed using Lipofectamine 2000 (Invitrogen, Thermo Fisher Scientific)-based method. 293T cells were transfected with vectors expressing either N or Nef^mut^/N, and supernatants harvested from 48 to 72 h after transfection. EVs were recovered through differential centrifugations by centrifuging supernatants at 500 × *g* for 10 min, and then at 10,000 × *g* for 30 min. Supernatants were harvested, filtered with 0.22 μm pore size filters, and ultracentrifuged at 70,000 × *g* for l h. Pelleted vesicles were resuspended in 1×PBS, and ultracentrifuged again at 70,000 × *g* for 1 h. Afterward, pellets containing EVs were resuspended in 1:100 of the initial volume.

Western blot analyses of both cell lysates and EVs were carried out as reported^11^, and filters were revealed using 1:2000 diluted anti-flag M2 mAb from Merck (cat. H1029), 1:500-diluted anti-β-actin mAb (cat. 122625, Cell Signaling), 1:500 diluted anti-Alix Abs (PA5-52873 Invitrogen). Filters were analyzed by a Chemi-Doc apparatus (Bio-Rad) and relevant signals quantified by Image Lab software version 6.1.

### Virus production

VERO-E6 cells were grown in DMEM (Gibco, Thermo Fisher) supplemented with 2% FCS, 100 units/mL penicillin, 100 μg/mL streptomycin, 2 mM L-glutamine, 1 mM sodium pyruvate, and non-essential amino acids (Gibco). The ancestral SARS-CoV-2/Italy INMI1#52284 viral isolate was propagated by inoculation of 70% confluent VERO-E6 cells^51^. Infected cell culture supernatant was harvested at 72 h post infection, clarified, aliquoted, and stored at −80 °C.

### Mouse infection

Before experimental infection, mice were anesthetized with a combination of ketamine (50 mg/kg of body weight) and medetomidine (1 mg/kg of body weight) administered intraperitoneally. A volume of 30 μL of each dilution was administered intranasally (i.n.), at 15 μL per nostril, dropwise. After virus challenge, intraperitoneal injection of atipamezole (1 mg/kg of body weight) was used as a reversal agent. For in vivo titration of the SARS-CoV-2/Italy INMI1#52284 isolate, age-matched K18-hACE2 mice were randomized by body weight into groups of 4, and challenged with 5-fold serial dilutions in 1×PBS of the virus preparation containing 2.2 × 10^5^, 4.4 × 10^4^, 8.8 × 10^3^, and 1.8 × 10^3^ TCID_50_ measured as previously reported^51^. As a negative control, 4 mice were sedated and an equal volume of 1×PBS was administered. Animals were assessed daily for clinical signs of infection. Virus challenge in immunized mice was performed using a virus dose of 4.4 lethal doses at 50% (LD_50_) resulting in a 99.99% predicted probability of mortality in untreated mice.

### Extraction and purification of lung RNA

Lungs were stored frozen after excision. After thawing, equal amounts of tissues were minced and incubated for 10 min in 1 mL of TRIzol™ Reagent (Thermo Fisher Scientific). Minced tissue was then passed through a QIAshredder homogenizer (Qiagen, Germantown, MD), and the flow-through was used for chloroform extraction according to the TRIzol™ protocol, using 0.2 mL of chloroform. Recovered total RNA was stored in water at −80 °C.

### RT-qPCR

RT-qPCR for SARS-CoV-2 Nucleocapsid (N) gene was performed using a One-Step Taqman-based strategy. Mouse β-actin amplification was included as loading control. All probes and primers (Table 1) were purchased from Integrated DNA Technologies (IDT). Each 20 µL reaction mixture contained 12 μL of qPCRBIO Probe 1-Step Virus Detect Lo ROX master mix (PCR Biosystems Ltd. London, UK), 3 µL of primers/probes mix, and of 1 µg of ezDNAse (Thermo Fisher)-treated RNA in a total of 5 µL. All samples were tested in duplicate, and samples with nuclease-free water alone were included as negative controls. Serial 10-fold dilutions of N1/N2 plasmid (10006625, 2019-nCoV_N_Positive Control from CDC, IDT, Belgium) were used to generate standard curves ranging from 1 to 100,000 copies. Median standard curve slope ranged from 3.25 to 3.43 with R^2^ > 0.998.

**Table 1:** Probes and primers used in RT-qPCR assays.

Gene name	Primer/Probe name	Sequence (5′–3′)
*SARS-CoV-2 N*	2019-nCoV_N2-P	FAM/ACAATTTGCCCCCAGCGCTTCAG/BHQ1
	2019-nCoV_N2-F	TTACAAACATTGGCCGCAAA
	2019-nCoV_N2-R	GCGCGACATTCCGAAGAA
*β-actin*	β-actin_P	FAM/ATTCCATAC/ZEN/CCAAGAAGGAAGGCTGG/3IABkFQ
	β-actin_ F	AGGTCTTTACGGATGTCAACG
	β-actin_R	ATTGGCAACGAGCGGTT

Samples were run on an Applied Biosystems 7500 Fast PCR system (Thermo Fisher). The following cycling conditions were applied: reverse transcription for 10 min at 55 °C followed by denaturation at 95 °C for 3 min. Then, 50 cycles of denaturation at 95 °C for 15 s and annealing/extension at 58 °C for 30 s. Amplification data were analyzed using Applied Biosystems 7500 software v2.3 (ThermoFisher). Results are reported as numbers of RNA copies for μg of total RNA.

### Statistical analysis

When appropriate, data are presented as mean ± standard error (s.e.m.). For the in vivo virus titration, virus dilutions and number of deaths/group after challenge were used to calculate the LD_50_ (Quest Graph™ LD_50_ Calculator, AAT Bioquest, Inc., S. Francisco, CA, USA). When indicated, the one- or two-tailed Mann–Whitney U test, the two-tailed Student T Test with Welch Correction, or the Kruskal–Wallis Test followed by Dunn’s Multiple Comparisons Test were conducted. Correlation analyses were conducted using the Spearman Rank Correlation Test. Statistical analyses were conducted with GraphPad Prism 9 software. *p* < 0.05 was considered statistically significant.

### Reporting summary

Further information on research design is available in the Nature Research Reporting Summary linked to this article.

## Supplementary information

Supplemental figures

REPORTING SUMMARY

## Data Availability

All data produced during the present study are available upon request made to the correspondence author.

## Materials availability

All requests for reagents should be directed to and will be fulfilled, upon the completion of a Material Transfer Agreement, by the correspondence author.
