# Supplementary material for: Antiviral effect of SARS-CoV-2 N-specific CD8+ T cells induced in lungs by engineered extracellular vesicles
Source: NPJ Vaccines. 2023 Jun 2;8:83. doi: 10.1038/s41541-023-00686-y (PMC10237059; doi:10.1038/s41541-023-00686-y)
Supplement: Supplementary file 1 — Supplemental figures [file 41541_2023_686_MOESM1_ESM.pdf]

Splenocytes

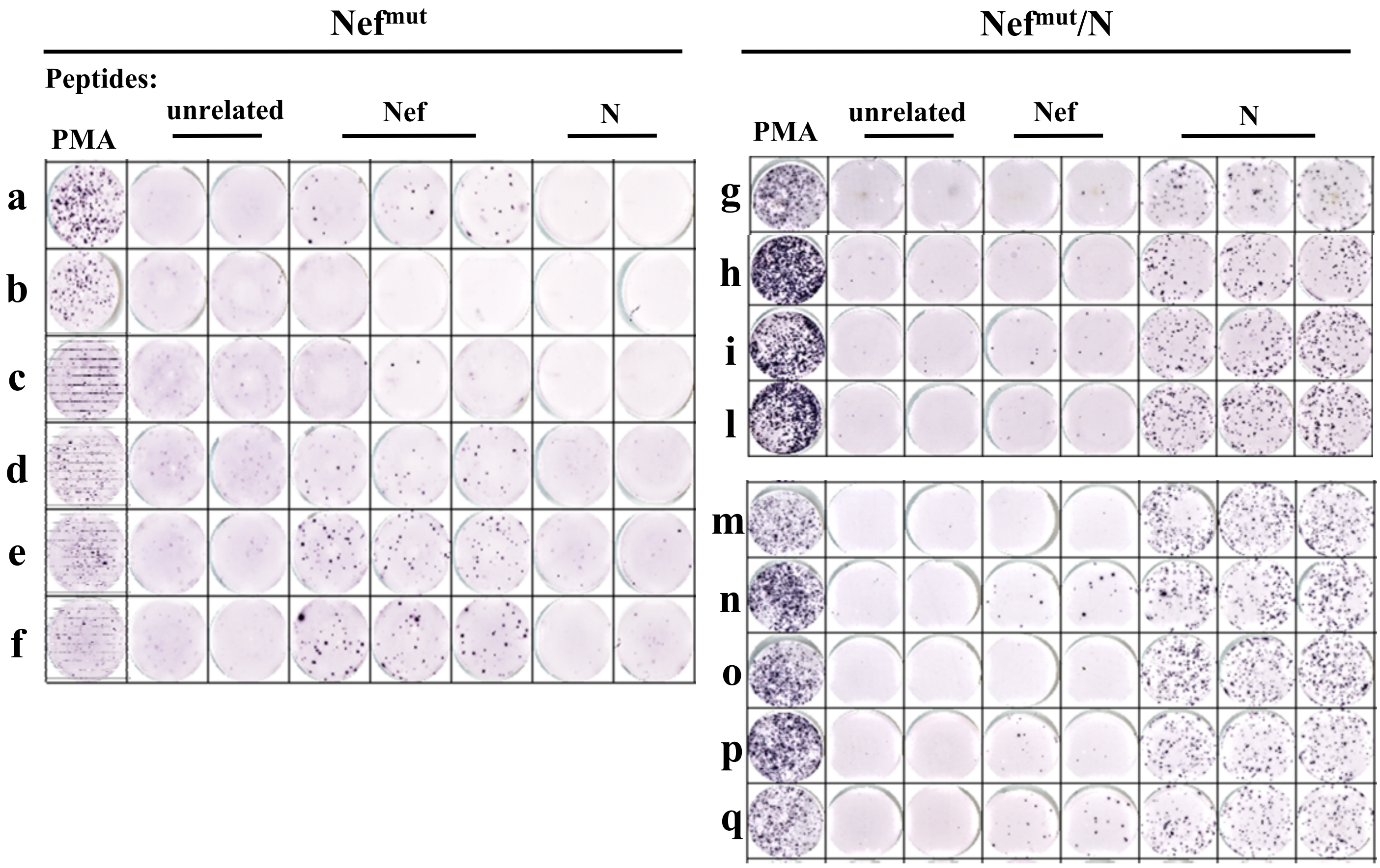

Supplementary fig. 1. Raw data from IFN-γ EliSpot analysis for the detection of SARS-CoV-2-N-specific $CD8^+$ T cells in splenocytes isolated from K18-hACE2 mice i.m. injected with either Nef^{mut}- (6 mice, a-f) or Nef^{mut}/N- (9 mice, g-q) expressing DNA vectors. A total of 2.5×10^5 splenocytes were incubated overnight with 5 µg/ml of either unrelated, Nef, or SARS-CoV-2-N specific peptides in IFN-γ EliSpot microwells. As a control, cells were also treated with PMA and ionomycin.

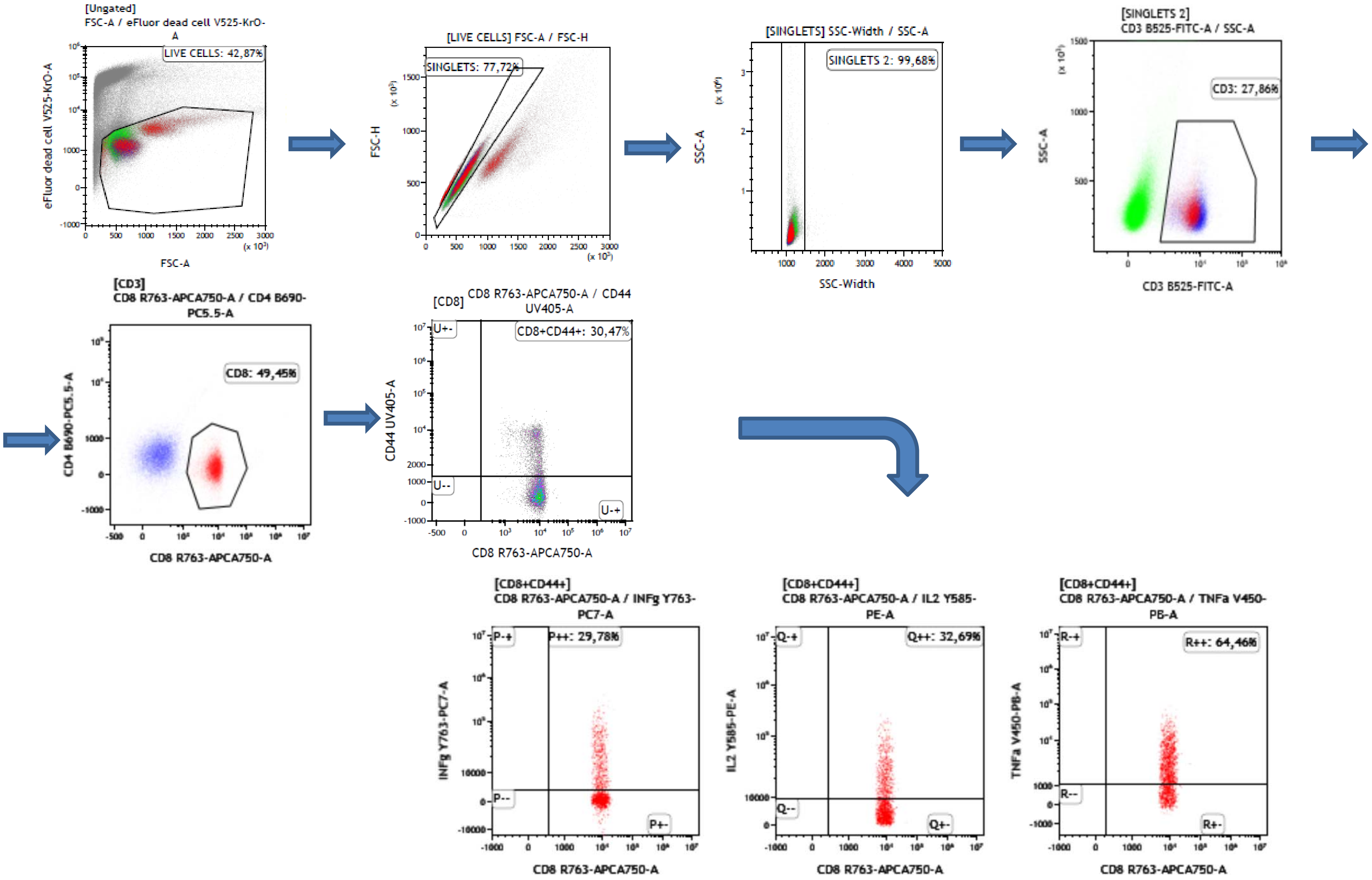

Supplementary fig. 2. Gating strategy carried out in ICS/flow cytometry analysis of splenocytes from injected mice. Shown is the analysis of PMA-treated cells.

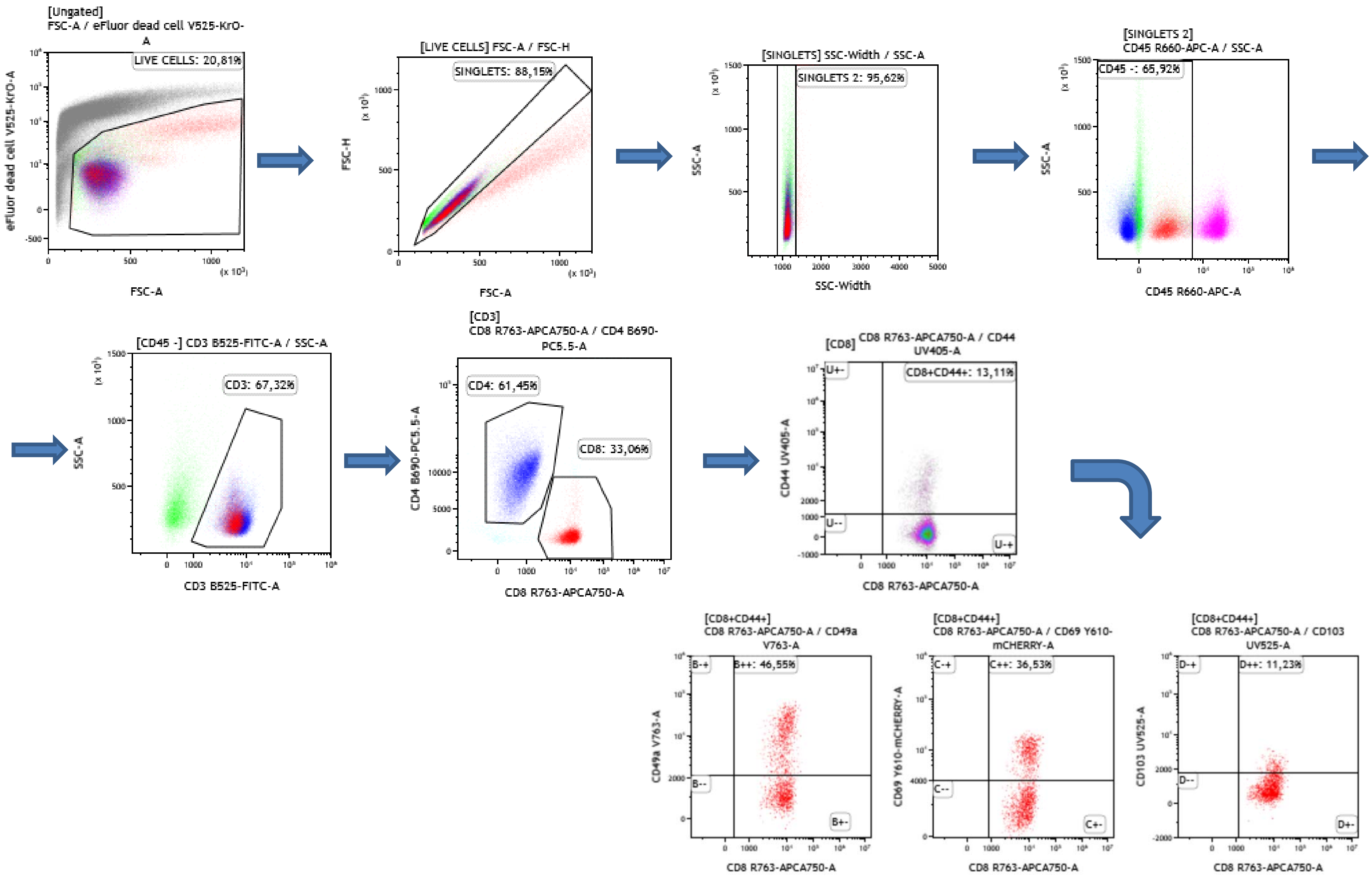

Supplementary fig. 3. Gating strategy carried out in ICS/flow cytometry analysis of CD45 negative immune cells isolated from lungs of injected mice. Shown is the analysis on cells treated with an unrelated peptide

CD45 mAb binding	Phenotype	Pool #1 (three mice)	Pool #2 (two mice)
+ (circulatory)	N-specific, polyfunctional $CD8^+$ T lymphocytes ($CD3^+$/$CD8^+$/$CD44^+$/IFN-γ^+/IL-2^+/TNFα^+)	0.35	0.74
- (lung)	N-specific, polyfunctional $CD8^+$ T lymphocytes ($CD3^+$/$CD8^+$/$CD44^+$/IFN-γ^+/IL-2^+/TNFα^+)	2.37	4.09
- (lung)	N-specific CD8+ Trm ($CD3^+$/$CD8^+$/$CD44^+$/$CD49a^+$/$CD69^+$/$CD103^+$/IFN-γ^+)	0.22	0.45

Supplementary fig. 4. Positive correspondence between percentages of circulatory and lung N-specific $CD8^+$ T lymphocytes in mice injected with Nef^{mut}/N DNA vector. K18-hACE2 mice (5 mice) were immunized with the Nef^{mut}/N DNA vector, and fifteen days after boosting, circulating blood cells were labeled with a fluorescently labeled anti-CD45 antibody in the tail vein 3 minutes before cervical dislocation. Immune cells were then isolated from lung tissues, and circulating cells were identified by flow cytometry as those labeled by the CD45 mAb, whereas the CD45 negative sub-population was considered as lung-associated immune cells.

ICS/flow cytometry analysis was performed on cells pooled from three (pool #1, low responders) or two (pool #2, high responders) immunized mice. N-specific $CD8^+$ T lymphocytes were scored in terms of polyfunctional (for both CD45 positive and negative cells) and Trm (for CD45 negative cells only) lymphocytes.

Shown are mean values of the percentages of each cell subpopulations from pooled cell cultures treated with specific peptides after subtraction of values detected in cultures treated with an unrelated peptide.

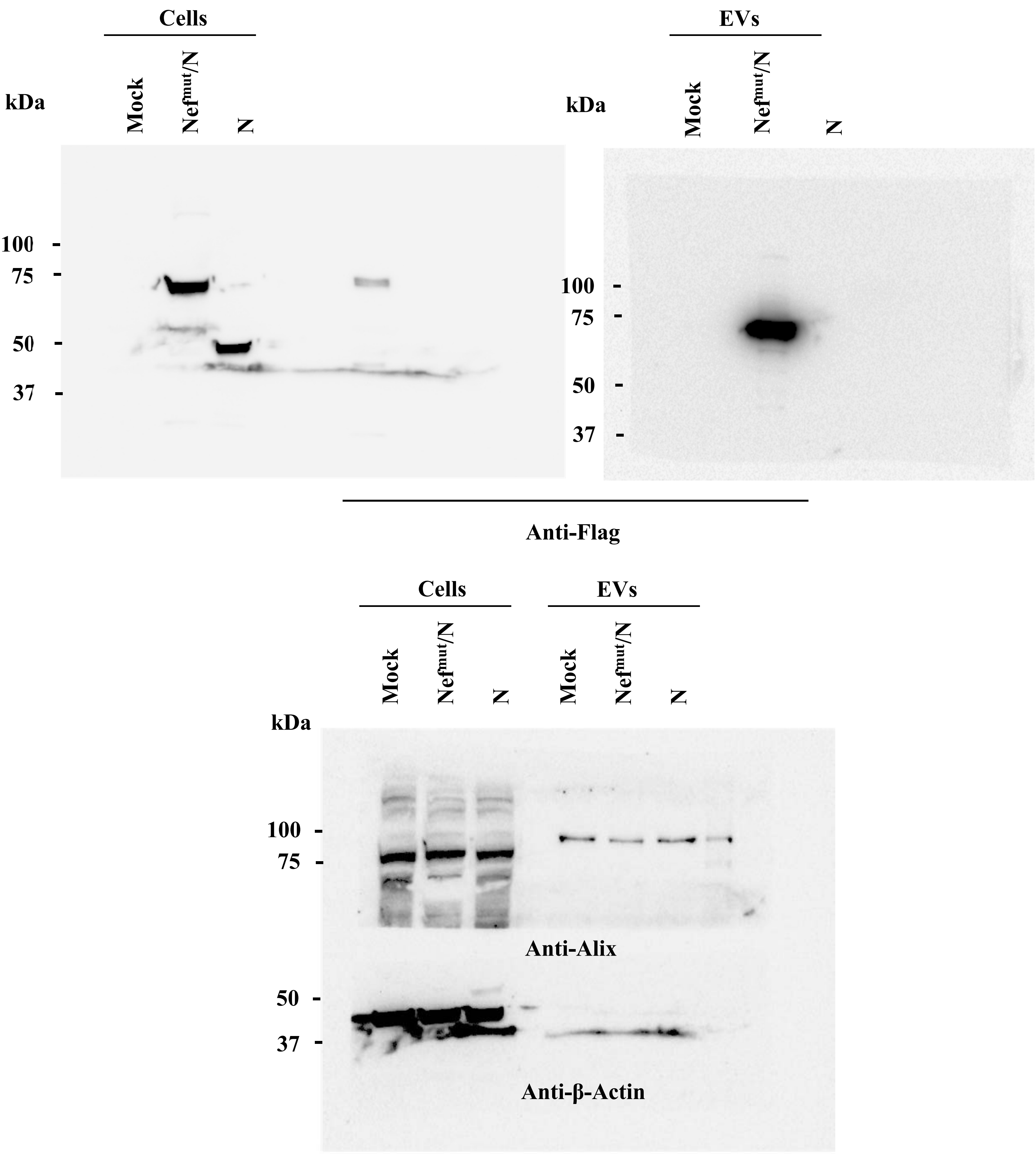

Supplementary fig. 5. Western blot analysis for the expression of Nef^{mut}/N and N in HEK293T cells and EVs isolated from the supernatants 48 hours after transfection with the respective DNA expression vectors. Raw data.
