# Supplementary material for: Antiviral effect of SARS-CoV-2 N-specific CD8+ T cells induced in lungs by engineered extracellular vesicles
Source: NPJ Vaccines. 2023 Jun 2;8:83. doi: 10.1038/s41541-023-00686-y (PMC10237059; doi:10.1038/s41541-023-00686-y)
Supplement: Supplementary file 2 — REPORTING SUMMARY [file 41541_2023_686_MOESM2_ESM.pdf]

nature portfolio

Corresponding author(s): NPJVACCINES-02827

Last updated by author(s): May 22, 2023

Reporting Summary

Nature Portfolio wishes to improve the reproducibility of the work that we publish. This form provides structure for consistency and transparency in reporting. For further information on Nature Portfolio policies, see our Editorial Policies and the Editorial Policy Checklist.

Statistics

For all statistical analyses, confirm that the following items are present in the figure legend, table legend, main text, or Methods section.

n/a	Confirmed	
☐	☒	The exact sample size (*n*) for each experimental group/condition, given as a discrete number and unit of measurement
☐	☒	A statement on whether measurements were taken from distinct samples or whether the same sample was measured repeatedly
☐	☒	The statistical test(s) used AND whether they are one- or two-sided<br>*Only common tests should be described solely by name; describe more complex techniques in the Methods section.*
☐	☒	A description of all covariates tested
☐	☒	A description of any assumptions or corrections, such as tests of normality and adjustment for multiple comparisons
☐	☒	A full description of the statistical parameters including central tendency (e.g. means) or other basic estimates (e.g. regression coefficient) AND variation (e.g. standard deviation) or associated estimates of uncertainty (e.g. confidence intervals)
☐	☒	For null hypothesis testing, the test statistic (e.g. *F*, *t*, *r*) with confidence intervals, effect sizes, degrees of freedom and *P* value noted<br>*Give P values as exact values whenever suitable.*
☒	☐	For Bayesian analysis, information on the choice of priors and Markov chain Monte Carlo settings
☒	☐	For hierarchical and complex designs, identification of the appropriate level for tests and full reporting of outcomes
☐	☒	Estimates of effect sizes (e.g. Cohen's *d*, Pearson's *r*), indicating how they were calculated

Our web collection on statistics for biologists contains articles on many of the points above.

Software and code

Policy information about availability of computer code

Data collection	Microsoft Power Point 2016; Microsoft Excel 2016; ICS/flow cytometry analysis: S CytoFLEX LX flow cytometer (Beckman Coulter); RT-qPCR: Applied Biosystems 7500 Fast PCR system (Thermo Fisher); IFN-gamma EliSpot: AELVIS ElisSpot reader (Hannover, Germany); Western blot images ChemiDoc imager (BioRad).
Data analysis	Microsoft Power Point 2016; Microsoft Excel 2016; ICS samples Kaluza Analysis Software 2.1 (Beckman Coulter); RT-qPCR, Applied Biosystems 7500 software v2.3 (ThermoFisher); IFN-gamma EliSpot: AELVIS ElisSpot reader (Hannover, Germany); Densitometry analysis: Bio-Rad Image Lab software 6.1 (BioRad), GraphPad Prism 9 software.

For manuscripts utilizing custom algorithms or software that are central to the research but not yet described in published literature, software must be made available to editors and reviewers. We strongly encourage code deposition in a community repository (e.g. GitHub). See the Nature Portfolio guidelines for submitting code & software for further information.

Data

Policy information about availability of data

All manuscripts must include a data availability statement. This statement should provide the following information, where applicable:

- Accession codes, unique identifiers, or web links for publicly available datasets
- A description of any restrictions on data availability
- For clinical datasets or third party data, please ensure that the statement adheres to our policy

All relevant results generated during the present study will be available upon request made to correspondence author

Human research participants

Policy information about studies involving human research participants and Sex and Gender in Research.

Reporting on sex and gender	N/A
Population characteristics	N/A
Recruitment	N/A
Ethics oversight	N/A

Note that full information on the approval of the study protocol must also be provided in the manuscript.

Field-specific reporting

Please select the one below that is the best fit for your research. If you are not sure, read the appropriate sections before making your selection.

☒ Life sciences ☐ Behavioural & social sciences ☐ Ecological, evolutionary & environmental sciences

For a reference copy of the document with all sections, see nature.com/documents/nr-reporting-summary-flat.pdf

Life sciences study design

All studies must disclose on these points even when the disclosure is negative.

Sample size	Sample size in animal studies was dictated by the 3R principle. In addition, already published results concerning immunogenicity/efficacy using the same immunogens helped us to minimize the number of mice included in each experimental group. Ex vivo assays were carried out with the maximum number of cells isolated from different animal tissues
Data exclusions	No data were excluded
Replication	The experimental design with animals is reported on fig. 1 of our ms. All cell samples isolated from animals were tested with numbers of replicates sufficient to calculate statistics as detailed in the figure legends. On the other hand, samples from lungs of immunized/infected animals were limited in terms of recovered number of cells. In some instances, this limitation hindered the possibility to produce a number of replications adequate for statistics
Randomization	K18-hACE2 mice were randomized by body weight
Blinding	In many instances, blinding was not possible in our studies. In animal experiments, blinding was precluded for safety reasons, considering the hazardous biological material we manipulated. In "ex vivo analysis", the paucity of cells isolated from lungs prevented the possibility to include the same number/type of negative control for each experimental condition.

Reporting for specific materials, systems and methods

We require information from authors about some types of materials, experimental systems and methods used in many studies. Here, indicate whether each material, system or method listed is relevant to your study. If you are not sure if a list item applies to your research, read the appropriate section before selecting a response.

Materials & experimental systems

n/a	Involved in the study	
☐	☒	Antibodies
☐	☒	Eukaryotic cell lines
☒	☐	Palaeontology and archaeology
☐	☒	Animals and other organisms
☒	☐	Clinical data
☒	☐	Dual use research of concern

Methods

n/a	Involved in the study	
☒	☐	ChIP-seq
☐	☒	Flow cytometry
☒	☐	MRI-based neuroimaging

Antibodies

Antibodies used

- APC-conjugated anti-mouse CD45 antibody, clone 104, cat. 20-04540U100, Tonbo-Bioscience
- anti-mouse CD16/CD32, clone 93, cat. 12-7021-82, eBioscience, Thermo Fisher
- FITC-conjugated anti-mouse CD3, clone 17A2, cat. 555274, BD Biosciences
- APC-Cy7-conjugated anti-mouse CD8a, clone53-6.7, cat. 557654, BD Biosciences
- PerCP conjugated anti-mouse CD4, clone RM4-5, cat. 553052, BD Biosciences
- BUV395-conjugated anti-mouse CD44, clone IM7, cat. 740215, BD Biosciences
- BUV750-conjugated anti-mouse CD49a, clone Ha31/8, cat. 746854, BD Biosciences
- PECF594-conjugated anti-mouse CD69, clone H1.2F3, cat. 562455, BD Biosciences
- BUV563-conjugated anti-mouse CD103, clone M290, cat. 741261, BD Biosciences
- PE-Cy7-conjugated anti-mouse IFN-γ, clone XMG1.2, cat. 25-7311-82, eBioscience, Thermo Fisher
- PE-conjugated anti-mouse IL-2, clone JES6-5H4, cat. 12-7021-82, eBioscience, Thermo Fisher
- BV421-conjugated anti-mouse TNF-α, clone MP6-XT22, cat. 563387, BD Biosciences
- Anti-mouse IFN-AN18 mAb, cat. 3321-3-250 (Mabtech)
- R4-6A2 biotinylated anti-IFN-γ, cat. 3321-6-250(Mabtech)
- anti-flag M2 mAb (H1029-Merck)
- anti-human β-actin monoclonal antibody, cat. 122625 (Cell signaling)
- HRP-conjugated anti-mouse IgG, cat. 170-6516 (BioRad)
- anti-human Alix polyclonal antibody, cat. PA5-52873 (Invitrogen)
- HRP-conjugated anti-rabbit IgG, cat. NA934V (Amersham)

Validation

All antibodies are commercially available and validated by the manufacturers

Eukaryotic cell lines

Policy information about cell lines and Sex and Gender in Research

Cell line source(s)

Human embryonic kidney (HEK) 293T cells (ATCC, CRL-11268). x vivo cells were obtained from female mice only.

Authentication

HEK293T cell authenticatiion was as indicated by ATCC

Mycoplasma contamination

HEK293T cells we used tested negative for mycoplasma, as assesed using PCR-based commercial assays.

Commonly misidentified lines
(See ICLAC register)

N/A

Animals and other research organisms

Policy information about studies involving animals; ARRIVE guidelines recommended for reporting animal research, and Sex and Gender in Research

Laboratory animals

Six-week-old C57 Bl/6 K18-hACE2 transgenic female mice

Wild animals

N/A

Reporting on sex

All mice we used were female. Sex was not considered in the study design.

Field-collected samples

The study did not involve samples collected from the field

Ethics oversight

Istituto Superiore di Sanità, approved by the Italian Ministry of Health, authorizations 565/2020 and 591/2021, released on June 3rd 2020 and July 30th 2021, respectively.

Note that full information on the approval of the study protocol must also be provided in the manuscript.

Flow Cytometry

Plots

Confirm that:

☒ The axis labels state the marker and fluorochrome used (e.g. CD4-FITC).

☒ The axis scales are clearly visible. Include numbers along axes only for bottom left plot of group (a 'group' is an analysis of identical markers).

☒ All plots are contour plots with outliers or pseudocolor plots.

☒ A numerical value for number of cells or percentage (with statistics) is provided.

Methodology

Sample preparation	PBMCs were recovered from EDTA-blood samples obtained through retro orbital puncture under topical anesthesia. Erythrocytes were removed by treatment with ACK lysing buffer (Gibco) according to the manufacturer's instructions.<br>To isolate splenocytes, spleens were explanted, placed into tubes containing 1 mL of RPMI 1640 and 50 µM 2-mercaptoethanol, then transferred into 60 mm Petri dishes with 2 mL of the same medium. Splenocytes were obtained by notching the spleen sac and pushing the cells out with the plunger seal of a 1 mL syringe. After addition of 2 mL of medium, cells were transferred into a 15 mL conical tube, and the Petri plate washed with 4 mL of medium to maximize cell recovery. Afterwards, cells were collected by centrifugation, resuspended in RPMI complete medium containing 50 µM 2-mercaptoethanol and 10% FCS, and counted.<br>For lung cell isolation, circulating blood cells were fluorescently labeled with 2 µg (10 µl of the commercial stock) of an anti-CD45 antibody (Tonbo-Bioscience, S. Diego, CA anti-mouse CD45.2-APC) diluted in 200 µl of 1×PBS inoculated in the tail vein exactly 3 minutes before cervical dislocation. For cell recovery, lungs were excised, washed with 1×PBS, cut into small pieces, and then digested for 30 minutes under gentle agitation at 37°C with 7 mL of 4 mg/mL of type III collagenase (Worthington Biochemical, Lakewood, NJ) and 0.05 mg/mL of DNase I (Sigma) in 1×PBS. After digestion, an equal volume of medium was added and cells were passed through a 70 µm cell strainer, washed, and resuspended in 1×PBS/ACK 1:1 for red blood cells lysis. Finally, the isolated cells were resuspended in complete culture medium and counted.
Instrument	CyotFLEX LX (Beckman Coulter, Brea, CA, USA)
Software	Kaluza software (Beckman Coulter).
Cell population abundance	Samples of at least 100,000 cells/condition were analyzed. Treatmet with fluorescently labelled anti-CD3 mAb served to identify the relevant cell populations to be analyzed
Gating strategy	Live cells as assessed by LIVE/DEAD dye vs. FSC-A, singlet cells from FSC-A vs. FSC-H (singlet 1) and SSC-A vs. SSC-W (singlet 2), CD3+ cells from CD3-FITC vs. SSC-A, CD8+, or CD4+ cells from CD8-APC-Cy7 vs. CD4-PerCP. CD3+/CD8+ cell population was gated against CD44+ cells, and, to detect polyfunctional CD8+ T lymphocytes, the population of cells positive for both CD8 and CD44 was analyzed for APC-Cy7, PE, and BV421 to detect simultaneous changes in IFN-γ, IL-2, and TNF-α production, respectively. To detect CD8+ T resident memory (Trm), the population of cells positive for both CD8 and CD44 was analyzed for the co-expression of CD49a, CD69, and CD103. Boolean gates were created to measure co-expression patterns.

☒ Tick this box to confirm that a figure exemplifying the gating strategy is provided in the Supplementary Information.
